# Achieving ultra-low contact barriers in MX_2_/SiH (M = Nb, Ta; X = S, Se) metal–semiconductor heterostructures: first-principles prediction†

**DOI:** 10.1039/d4na00482e

**Published:** 2024-07-26

**Authors:** Son T. Nguyen, Chuong V. Nguyen, Huynh V. Phuc, Nguyen N. Hieu, Cuong Q. Nguyen

**Affiliations:** a Faculty of Electrical Engineering, Hanoi University of Industry Hanoi 100000 Vietnam nguyensontung@haui.edu.vn; b Department of Materials Science and Engineering, Le Quy Don Technical University Hanoi 100000 Vietnam chuong.vnguyen@lqdtu.edu.vn; c Division of Physics, School of Education, Dong Thap University Cao Lanh 870000 Vietnam; d Institute of Research and Development, Duy Tan University Da Nang 550000 Vietnam nguyenquangcuong3@duytan.edu.vn; e Faculty of Natural Sciences, Duy Tan University Da Nang 550000 Vietnam

## Abstract

Minimizing the contact barriers at the interface, forming between two different two-dimensional metals and semiconductors, is essential for designing high-performance optoelectronic devices. In this work, we design different types of metal–semiconductor heterostructures by combining 2D metallic MX_2_ (M = Nb, Hf; X = S, Se) and 2D semiconductor SiH and investigate systematically their electronic properties and contact characteristics using first principles calculations. We find that all the MX_2_/SiH (M = Nb, Ta; X = S, Se) heterostructures are energetically stable, suggesting that they could potentially be synthesized in the future. Furthermore, the generation of the MX_2_/SiH metal–semiconductor heterostructures leads to the formation of the Schottky contact with ultra-low Schottky barriers of a few tens of meV. This finding suggests that all the 2D MX_2_ (M = Nb, Ta; X = S, Se) metals act as effective electrical contact 2D materials to contact with the SiH semiconductor, enabling electronic devices with high charge injection efficiency. Furthermore, the tunneling resistivity of all the MX_2_/SiH (M = Nb, Ta; X = S, Se) MSHs is low, confirming that they exhibit high electron injection efficiency. Our findings underscore fundamental insights for the design of high-performance multifunctional Schottky devices based on the metal–semiconductor MX_2_/SiH heterostructures with ultra-low contact barriers and high electron injection efficiency.

## Abstract 2

Minimizing the contact barriers at the interface, forming between two different two-dimensional metals and semiconductors, is essential for designing high-performance optoelectronic devices.

## Graphical abstract

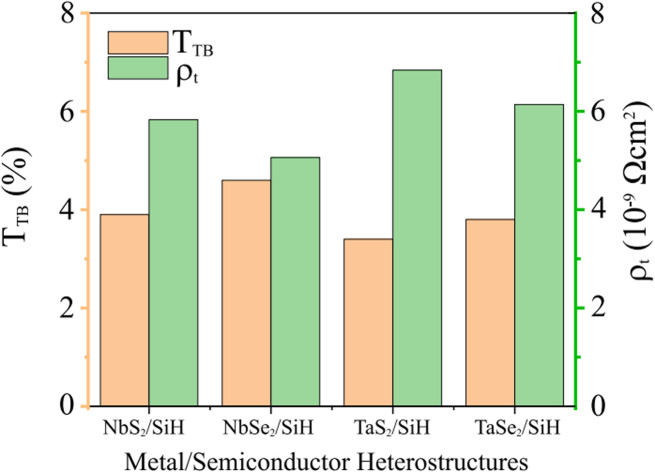

## 1 Introduction

Recently, two-dimensional (2D) materials, including graphene,^1^ transition metal dichalcogenides (TMDCs),^2^ phosphorene^3^ and MXenes,^4^ have garnered significant interest in the scientific community owing to their intriguing physical properties. Among these 2D materials, considerable attention has recently been directed towards TMDCs owing to their versatility in physical properties.^5^ Most TMDC 2D materials, such as MoS_2_,^2^ MoSe_2_,^6^ WS_2_,^7^ and WSe_2_,^8^ exhibit semiconducting characteristics. Unlike these semiconductors, NbS_2_, NbSe_2_, TaS_2_ and TaSe_2_ monolayers exhibit metallic behaviors. Recently, it has been discovered that these 2D metals serve as effective electrodes when integrated with other 2D semiconductors, facilitating the creation of metal/semiconductor heterostructures (MSHs) with significantly reduced contact barriers.^9–12^ For instance, Fu *et al.*^9^ successfully grew high-quality and clean NbS_2_/MoS_2_ MSH *via* one-step chemical vapor deposition (CVD). Tsoutsou *et al.*^11^ demonstrated that the TaSe_2_ monolayer could form low barrier contacts with other semiconductors, including HfSe_2_ and MoSe_2_ due to the small difference in their work functions. Using first-principles prediction, Nguyen *et al.*^12^ found that integrating 2D metallic TaSe_2_ with semiconducting WSe_2_ monolayers leads to the creation of TaSe_2_/WSe_2_ MSH with small resistivity, having great potential for the fabrication of novel Schottky devices. The search for appropriate 2D semiconductors to contact with 2D MX_2_ (M = Nb, Ta; X = S, Se) monolayers is intensifying in recent years as researchers aim to uncover ideal combinations for enhancing electronic and optoelectronic device applications.

More recently, silicane (SiH), a novel 2D material has been predicted by full hydrogenation of monolayer silicene on both sides.^13,14^ It should be noted that silicane can also be synthesized by mechanical exfoliation, a strategy that has been used to obtain germanane (GeH).^15^ Additionally, hydrogenation opens a band gap, stabilizes the structure and eliminates conductivity in the silicene monolayer.^16^ Moreover, the electronic, thermoelectric and optical properties of the SiH monolayer are sensitive to external conditions, including strains,^17^ electric fields^18^ and constructing heterostructures.^19–22^ Among those strategies, construction of heterostructures has been proven to be one of the most effective strategies to enhance the physical properties of the SiH monolayer. For instance, we previously investigated the electronic properties and carrier mobility of the BP/SiH heterostructure as well as the effect of an electric field. The combination of SiH and BP monolayers gives rise to an enhancement in the optical absorption and carrier mobility compared to the constituent monolayers. Sheng *et al.*^23^ combined the InSe/SiH heterostructure and demonstrated that such combination leads to an enhancement of photocatalytic efficiency. Furthermore, the combination between SiH and PtSe_2_ (ref. 24) or AlAs^25^ also leads to an enhancement in the absorption coefficient and photocatalytic properties. It is found that the physical properties of the SiH material can be tuned when it is combined with other 2D semiconductors. However, to date, the combination between SiH and other 2D metals has not yet been extensively investigated.

In this work, we design MX_2_/SiH MSHs (M = Nb, Ta; X = S, Se) by stacking the 2D MX_2_ (M = Nb, Ta; X = S, Se) metals above on top of the SiH semiconductor using first-principles calculations. It is demonstrated that all the 2D MX_2_/SiH (M = Nb, Ta; X = S, Se) metal–semiconductor heterostructures form the Schottky contact with ultra-low contact barriers of a few tens of meV. Our findings could provide fundamental insights and open an avenue for the design of high-performance multifunctional Schottky devices based on the metal–semiconductor MX_2_/SiH heterostructures with ultra-low contact barriers and high electron injection efficiency.

## 2 Computational methods

Our calculations, including geometric optimization and the calculations of the interface properties, were carried out using density functional theory (DFT) as implemented in the simulation Quantum Espresso package.^26^ The electron exchange and correlation energy were described using the generalized gradient approximation (GGA) within the Perdew–Burke–Ernzerhof (PBE) formulation.^27^ For more accurate results, the hybrid Heyd–Scuseria–Ernzerhof (HSE06) functional was employed to calculate the band gap of the 2D semiconductor.^28^ Furthermore, the long-range interactions that may exist in layered 2D materials were described by adding the DFT-D3 method.^29^ A vacuum thickness of 27 Å was added along the thickness of the heterostructures to avoid any spurious interactions. All atomic positions were relaxed until the energy and forces converged to 10^−6^ eV and 0.01 eV Å^−1^, respectively. The cut-off energy of 520 eV and a *k*-point mesh of 9 × 9 × 1 were used for all the calculations. It should be noted that the cut-off energy was gradually increased until the total energy converged to within a tolerance of less than 0.01 eV Å^−1^. Whereas, we used such *k*-point grids that balanced accuracy and computational cost, ensuring that the electronic structure calculations remained precise with changes in total energy below 0.01 eV Å^−1^.

## 3 Results and discussion

**Fig. 1:**
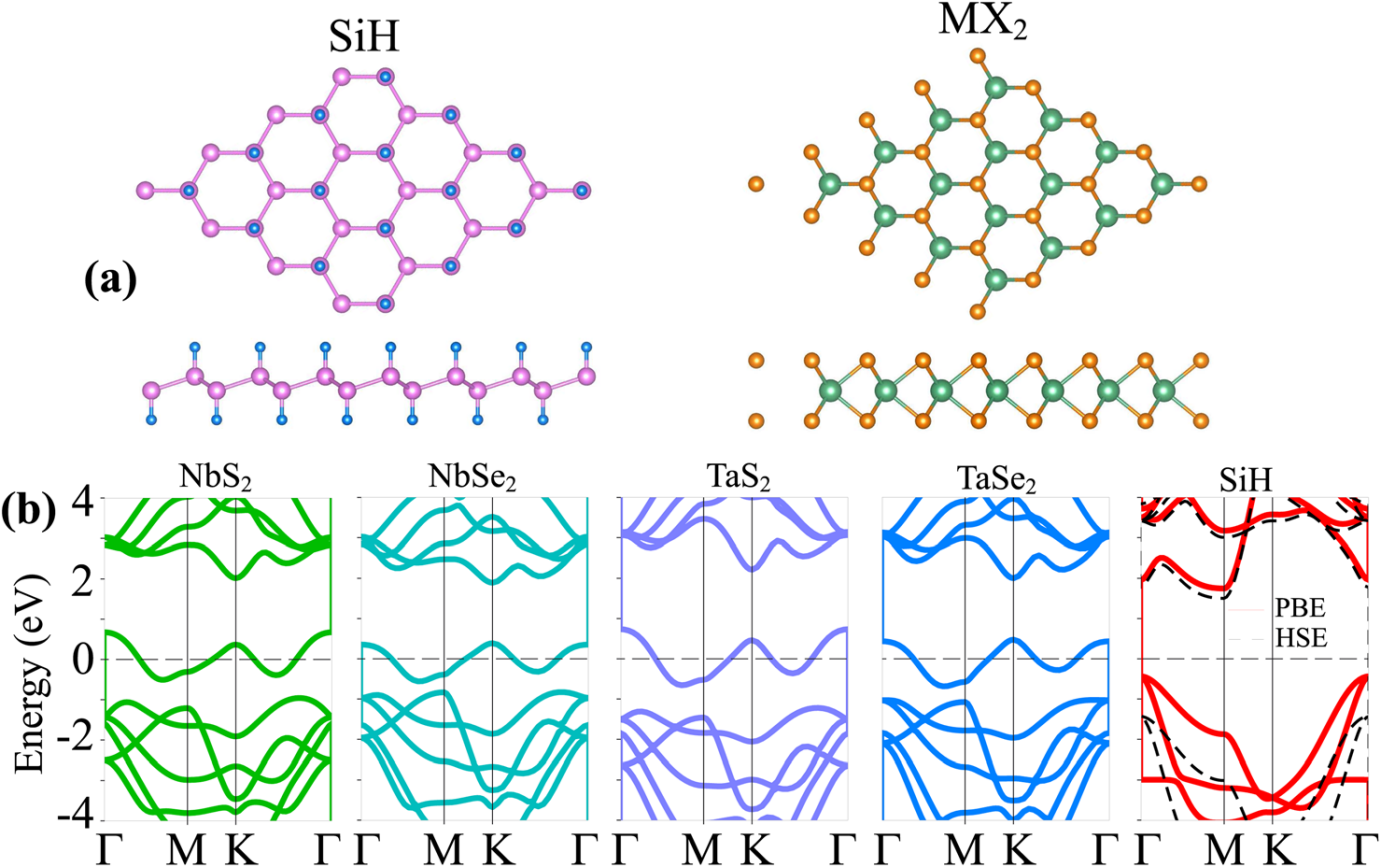
(a) Top and side views of the atomic structures and (b) band structures of monolayers SiH and MX_2_ (M = Nb, Ta; X = S, Se). The Fermi level is set to be zero.

We first examine the atomic structures and electronic properties of 2D MX_2_ (M = Nb, Ta; X = S, Se) metals and the SiH semiconductor. The atomic structures and band structures of these 2D materials are depicted in Fig. 1. The SiH monolayer exhibits a hexagonal atomic arrangement with a buckled structure, as illustrated in Fig. 1(a). The hydrogen atoms are passivized on both sides of silicon atoms. The lattice constant of the SiH monolayer is obtained as 3.86 Å, which is consistent with previous reports.^18,23^ Similarly, the MX_2_ (M = Nb, Ta; X = S, Se) monolayers also possess a hexagonal structure. One transition M (M = Nb, Ta) metal is sandwiched between two different chalcogenide X (X = S, Se) atoms on both sides. The lattice constants of NbS_2_, NbSe_2_, TaS_2_ and TaSe_2_ monolayers are calculated to be 3.31, 3.43, 3.31 and 3.44 Å, respectively. These values are also close to the experimental measurements and theoretical reports. Furthermore, we investigate the band structures of all the MX_2_ (M = Nb, Ta; X = S, Se) metals and the SiH semiconductor, as illustrated in Fig. 1(b). All the MX_2_ materials exhibit metallic characteristics with the band crossing the Fermi level. On the other hand, the SiH monolayer shows semiconducting behavior with an indirect band gap. The maxima of valence bands (VBM) and minima of conduction bands (CBM) of the SiH monolayer are located at the *Γ* and *M* point, respectively. The band gap of SiH is predicted to be 2.18 and 2.96 eV, as measured using PBE and HSE approaches, respectively. Moreover, the Fermi level of the SiH semiconductor is closer to the VBM than the CBM, indicating that the SiH monolayer is a p-type extrinsic semiconductor. Our results are in good agreement with previous predictions.^25,30^

**Fig. 2:**
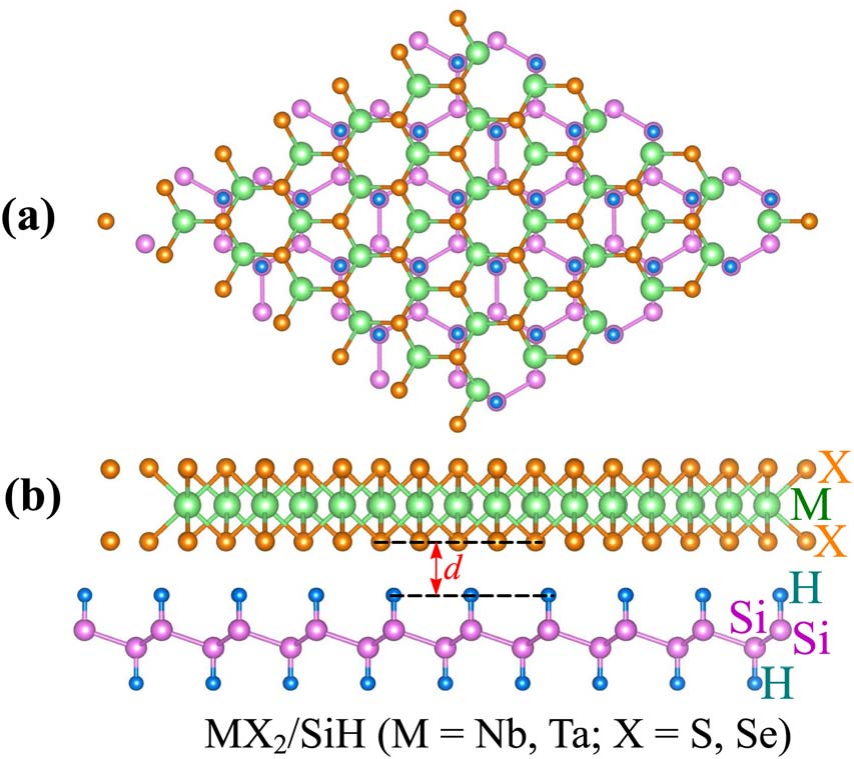
(a) Top view and (b) side views of the atomic structures of the MX_2_/SiH MS-vdWHs (M = Nb, Ta; X = S, Se).

We further design the MX_2_/SiH MSHs by vertically stacking the 2D MX_2_ metals above on top of the 2D SiH semiconductor. The atomic structures of the MX_2_/SiH MSHs are depicted in Fig. 2 and S1 of the ESI.† We investigated four different stacking patterns (SP) of the MX_2_/SiH MSHs. The most energetically favorable SP is depicted in Fig. 2. To minimize the effects of strain caused by the lattice mismatch between the two different layers, the MX_2_/SiH MSHs are designed by using (2 × 2) and 
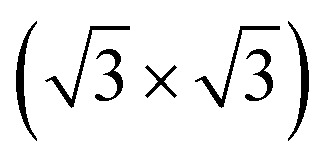
 supercells of MX_2_ and SiH layers, respectively. The overall lattice mismatch for the MX_2_/SiH MSHs is less than 2%. This value is still small and affects insignificantly the electronic properties of these 2D materials. After geometric optimization, the interlayer distances between the MX_2_ and SiH layers are obtained and listed in Table 1. The interlayer distances vary from 2.31 Å for the TaS_2_/SiH to 2.33 Å for the NbS_2_/SiH and to 2.37 Å for the TaSe_2_/SiH and to 2.39 Å for the NbSe_2_/SiH MSHs. This finding indicates that the interlayer distances in Nb(Ta)S_2_/SiH are shorter than those in the Nb(Ta)Se_2_/SiH MSH. In addition, we can find that these values of the interlayer distances in MX_2_/SiH heterostructures are comparable to those in other SiH-based heterostructures, such as AlAs/SiH,^25^ SiH/CdI_2_ (ref. 31) and InSe/SiH.^23^ Furthermore, we check the stability of all the MX_2_/SiH MSHs by calculating the binding energy as follows:1
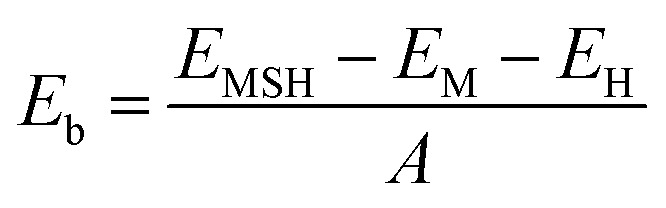
Here, *E*_MSH_, *E*_M_ and *E*_H_ are the total energies of the heterostructure, isolated MX_2_ metal and the SiH semiconductor, respectively. The binding energies of all the MX_2_/SiH MS-vdWHs (M = Nb, Ta; X = S, Se) MSHs are listed in Table 1. These values are comparable with those in other combined heterostructures.^23,32^ In addition, a negative binding energy suggests that all the MX_2_/SiH MS-vdWHs (M = Nb, Ta; X = S, Se) MSHs are energetically stable, suggesting that these MSHs could potentially be synthesized in the future *via* epitaxial growth^33^ or chemical vapor deposition.^9,34^

**Table 1:** Calculated lattice constant (*a*, Å), interlayer distance (*d*, Å), binding energy (*E*_b_, meV Å^−2^), contact barriers *Φ*_p_ and *Φ*_n_, eV and contact type in the MX_2_/SiH MSHs (M = Nb, Ta; X = S, Se) MSHs

	*a*, Å	*d*, Å	*E*_b_, meV Å^−2^	*Φ*_p_, eV	*Φ*_n_, eV	Contact types
NbS_2_/SiH	6.62	2.33	−14.50	0.010	2.15	p-ShC
NbSe_2_/SiH	6.80	2.39	−12.61	0.024	2.15	p-ShC
TaS_2_/SiH	6.63	2.32	−14.17	0.014	2.14	p-ShC
TaSe_2_/SiH	6.81	2.37	−12.72	0.067	2.12	p-ShC

**Fig. 3:**
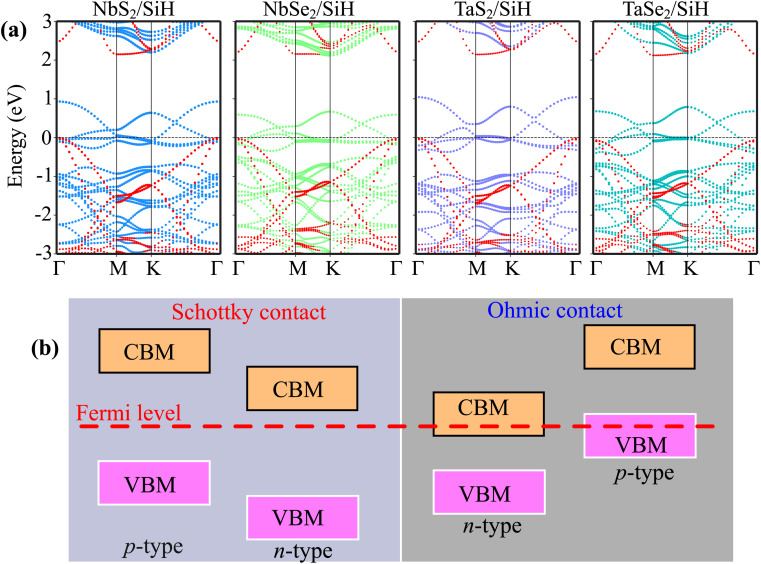
(a) Projected band structures of the MX_2_/SiH MS-vdWHs (M = Nb, Ta; X = S, Se) and (b) the schematic model of the band alignment in MS-vdWHs. Red, cyan, green, purple and dark-blue circles represent the contributions of the SiH, NbS_2_, NbSe_2_, TaS_2_ and TaSe_2_ layers, respectively.

The projected band structures of the MX_2_/SiH (M = Nb, Ta; X = S, Se) MSHs are depicted in Fig. 3(a). One can find that the band structures of the MX_2_/SiH MSHs appear to be a sum of the band structures of the constituent MX_2_ metal and SiH semiconductor. More interestingly, the combination between MX_2_ metals and the SiH semiconductor leads to generation of metal/semiconductor heterostructures. Depending on the position of the band edges of the semiconductor relative to the Fermi level of metal, the combined heterostructure can form either the Schottky contact (ShC) or ohmic contact (OhC), as illustrated in Fig. 3(b). By analyzing the projected band structures, we observe that all the MX_2_/SiH (M = Nb, Ta; X = S, Se) MSHs lead to generation of the Schottky contact. In the band structures of the MX_2_/SiH (M = Nb, Ta; X = S, Se) MSHs, the Fermi level of the MX_2_ metal lies between the band edges of the SiH semiconductor. In addition, we find that the VBM of the SiH semiconductor is closer to the Fermi level than its CBM, indicating that all the MX_2_/SiH (M = Nb, Ta; X = S, Se) MSHs exhibit the p-type Schottky contact. The Schottky contact barriers for the p-type and n-type Schottky contact (ShC) can be obtained as: *Φ*_p_ = *E*_F_ – *E*_VBM_ and *Φ*_n_ = *E*_CBM_ – *E*_F_, where *E*_VBM_ and *E*_CBM_, respectively, are the energy of the VBM and CBM of the semiconductor SiH. *E*_F_ is the Fermi level of the MX_2_/SiH heterostructure. The contact barriers for the MX_2_/SiH MSHs are listed in Table 1. It is interesting that the *p*-type Schottky barriers in all the MX_2_/SiH (M = Nb, Ta; X = S, Se) MSHs are ultra-low. The NbS_2_/SiH MSH shows the smallest p-type Schottky barrier of 10 meV, while the largest Schottky barrier is observed in the TaSe_2_/SiH MSH, which is only 67 meV. The observed ultra-low contact barrier in the MX_2_/SiH (M = Nb, Ta; X = S, Se) MSHs indicates that the charge injection efficiency in these heterostructures is highly advantageous.^35,36^ Thereby, the 2D MX_2_ metals act as effective electrical contact 2D materials to contact with the SiH semiconductor, enabling electronic devices with high charge injection efficiency.

Furthermore, to examine the charge injection efficiency of all the MX_2_/SiH MSHs, we calculate the tunneling probability and contact tunneling resistivity as follows:^37^2
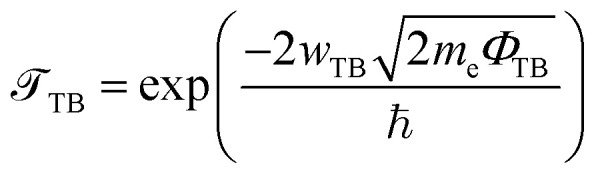
and
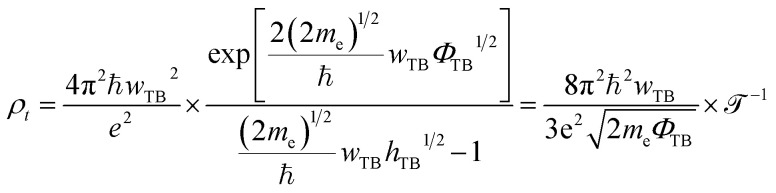


**Fig. 4:**
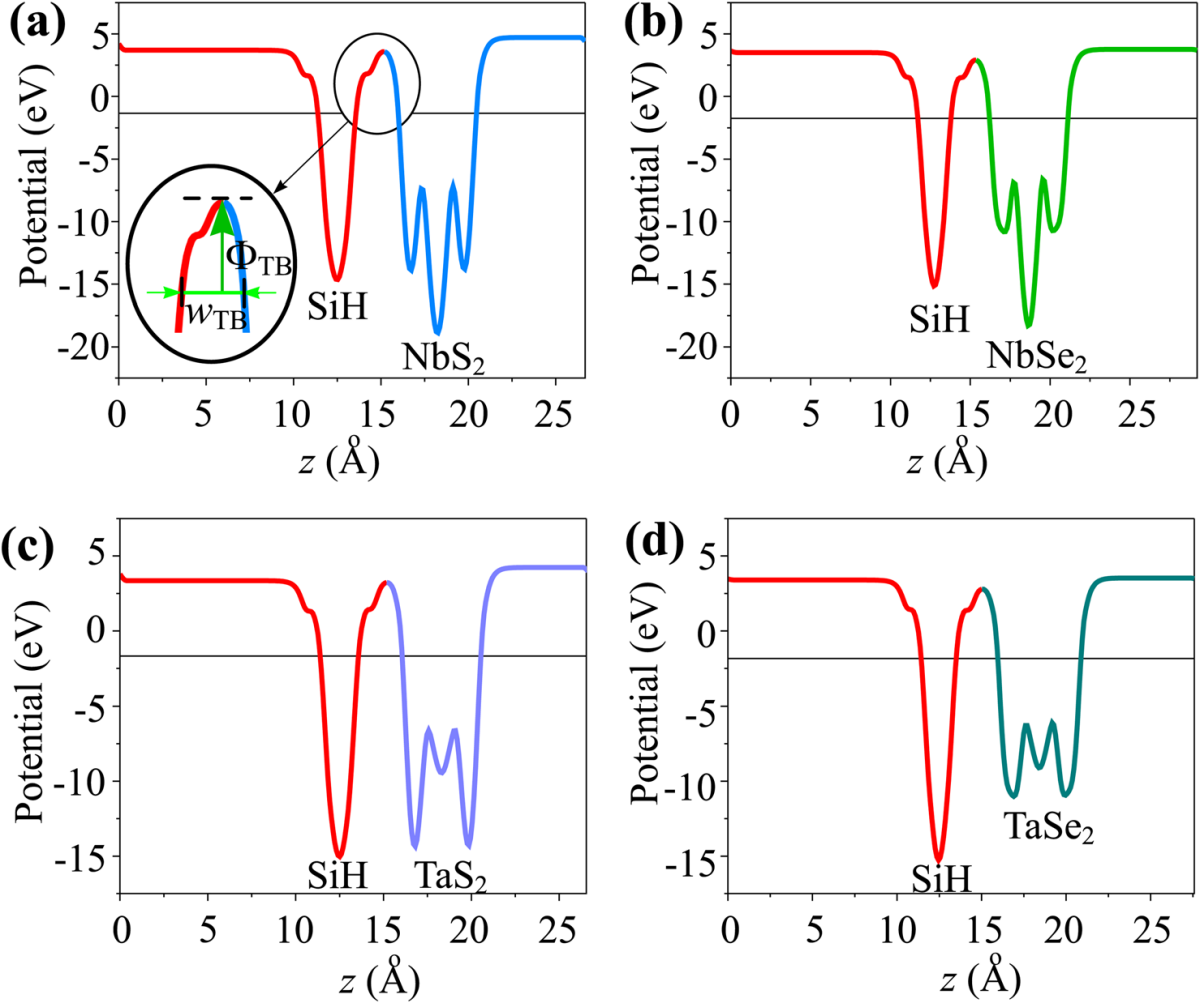
Calculated electrostatic effective potentials of (a) NbS_2_/SiH, (b) NbSe_2_/SiH, (c) TaS_2_/SiH and (d) TaSe_2_/SiH MSHs.

**Fig. 5:**
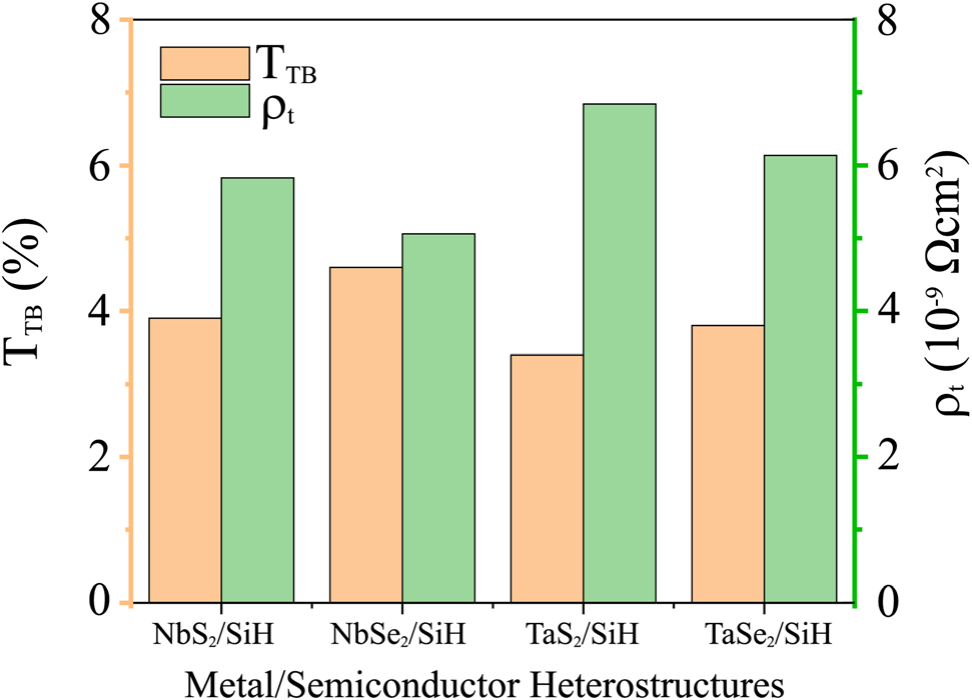
Calculated tunneling probability and contact tunneling resistivity of all the MX_2_/SiH (M = Nb, Ta; X = S, Se) MSHs.

The *w*_TB_ and *Φ*_TB_ represent the tunneling width and tunneling height, which can be obtained by analyzing the electrostatic potential of the considered heterostructures. The electrostatic potentials of all the MX_2_/SiH (M = Nb, Ta; X = S, Se) MSHs are depicted in Fig. 4. The obtained tunneling probability and contact tunneling resistivity of all the MX_2_/SiH (M = Nb, Ta; X = S, Se) MSHs are listed in Table 2 and illustrated in Fig. 5. One can find that a higher tunneling probability is correlated with lower tunneling resistivity^38^ and enhanced electron injection. The tunneling probability of the NbSe_2_/SiH MSH is higher than that of the other heterostructures, suggesting that the NbSe_2_ 2D metal acts as a superior electrical contact 2D material to contact with the SiH semiconductor to achieve high charge injection efficiency. Additionally, we find that the tunneling resistivity of all the MX_2_/SiH (M = Nb, Ta; X = S, Se) MSHs is as low as that of the low-contact-resistance Bi/MoS_2_.^39,40^ A lower tunneling resistivity correlates with a higher electron injection efficiency. Therefore, the electron injection efficiency in all the MX_2_/SiH MSHs is high. These findings suggest that the MX_2_ 2D metals can act as promising electrodes for sub-10 nm field-effect transistors.^41^ Additionally, the work functions of NbS_2_, NbSe_2_, TaS_2_ and TaSe_2_ 2D metals are calculated to be 6.12, 5.90, 5.51 and 5.36 eV. It is obvious that the work function of the MX_2_ monolayers is still higher than that for the common electrode graphene (4.6 eV).^42^ The large value of the work functions in the MX_2_ 2D metals leads to an alignment towards the VBM of the 2D SiH semiconductor, forming a p-type Schottky contact.^43^

**Table 2:** Calculated tunneling barrier width (*w*_TB_) and height (*Φ*_TB_), tunneling probability 
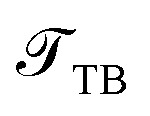
 and contact tunneling resistivity of all the MX_2_/SiH (M = Nb, Ta; X = S, Se) MSHs

	*w*_TB_, Å	*Φ*_TB_, eV	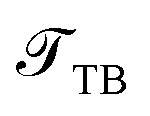 , %	*ρ*_t_, 10^−9^ Ω cm^2^
NbS_2_/SiH	3.57	3.73	3.90	5.83
NbSe_2_/SiH	2.90	4.17	4.60	5.06
TaS_2_/SiH	3.23	4.15	3.40	6.84
TaSe_2_/SiH	2.78	4.32	3.80	6.14

In addition, as illustrated in Fig. 4, we can see that the potential of the SiH layer is higher than that of the NbS(Se)_2_ layer in their corresponding MSHs, but it is deeper than the potential of the TaS(Se)_2_ layer. The charges flow from the layer with a deeper potential to the layer with a higher one. Therefore, electrons are transferred from the NbS(Se)_2_ layer to the SiH layer in the NbS(Se)_2_/SiH MSH. On the other hand, charges will flow from SiH to the TaS(Se)_2_ layer in the TaS(Se)_2_/SiH MSH.

We further calculated the charge density difference (CDD) in the MX_2_/SiH (M = Nb, Ta; X = S, Se) MSHs to visualize the charge redistribution and charge transfer between the two constituent monolayers. The CDD in the heterostructure can be obtained from the difference in the charge densities of the heterostructure (*ρ*_MSH_) and the constituent metal (*ρ*_M_) and semiconductor (*ρ*_S_) as follows:

3 Δ*ρ* = *ρ*_MSH_ − *ρ*_M_ − *ρ*_S_

The amount of charge transfer at the interface of MX_2_/SiH MSHs is obtained as:^44,45^4
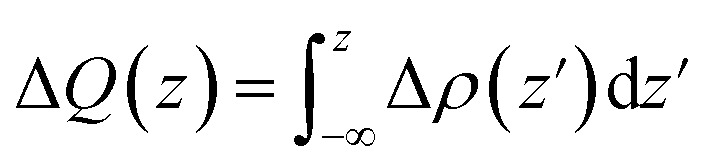


**Fig. 6:**
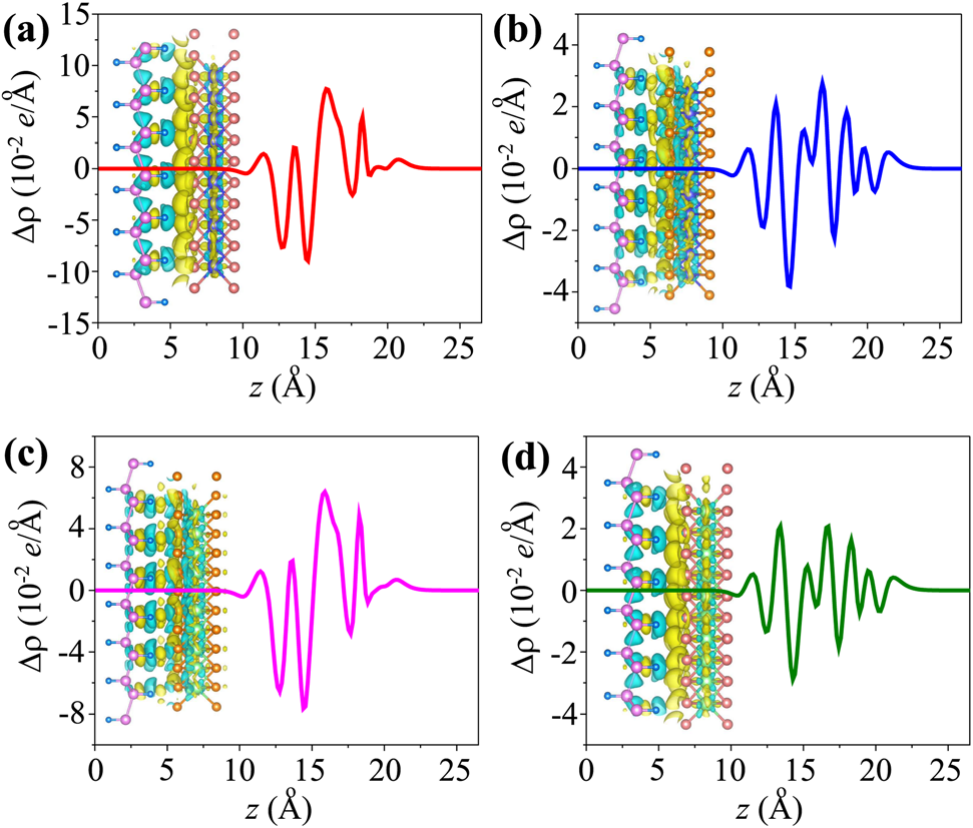
The planar-averaged CDD of the (a) NbS_2_/SiH, (b) NbSe_2_/SiH, (c) TaS_2_/SiH and (d) TaSe_2_/SiH MSHs. Yellow/cyan indicates the contribution of the charge accumulation/depletion. The insets show the 3D isosurfaces of the MSHs.

The CDD for all the MX_2_/SiH (M = Nb, Ta; X = S, Se) MSHs is depicted in Fig. 6. Yellow and cyan regions indicate positive and negative charges, respectively. For the NbS_2_/SiH heterostructure, the positive charges are mainly accumulated in the NbS_2_ layer, while the negative charges are depleted in the SiH layer, as depicted in Fig. 6(a) and its inset. This finding indicates that the electrons are transferred from the NbS_2_ layer to the SiH layer, *i.e.* the metallic NbS_2_ layer loses electrons, while the semiconducting SiH layer gains them. A similar trend of charge transfer is also observed in the NbSe_2_/SiH MSH, as illustrated in Fig. 6(b). On the other hand, for the TaS(Se)_2_/SiH MSH, the positive charges are mainly accumulated on the side of the SiH layer, while the negative charges are depleted in the TaS(Se)_2_ layer. The electrons are transferred from the SiH layer to the TaS(Se)_2_ layer. From eqn (5), the amount of charge transfer at the interfaces of the NbS_2_/SiH, NbSe_2_/SiH, TaS_2_/SiH and TaSe_2_/SiH heterostructures is calculated to be 0.045, 0.012, 0.024 and 0.015*e*, respectively. Such amount of charge transfer is small, indicating that weak interactions are dominant at the interface of heterostructures. Furthermore, it should be noted that owing to the ultra-low Schottky barriers, the contact barriers and contact types in the MX_2_/SiH MSHs can be easily tuned by external conditions.^46–51^ For instance, applying a compressive strain of −3% includes a transition from Schottky to the ohmic contact in the NbS_2_/SiH heterostructure, as illustrated in Fig. S2 of the ESI.† The versatility in the contact behavior of the MX_2_/SiH MSHs under external conditions makes them promising candidates for next-generation multifunctional devices.

## 4 Conclusions

In summary, we have investigated the atomic structures, electronic properties and the formation of ultra-low contact barriers in the MX_2_/SiH (M = Nb, Ta; X = S, Se) MSHs using first-principles calculations. All the MX_2_/SiH (M = Nb, Ta; X = S, Se) MSHs are energetically stable, suggesting that these MSHs could potentially be synthesized in the future. More interestingly, the generation of the MX_2_/SiH metal–semiconductor heterostructures leads to the formation of the Schottky contact with ultra-low Schottky barriers of a few tens of meV. This finding suggests that all the 2D MX_2_ (M = Nb, Ta; X = S, Se) metals act as effective electrical contact 2D materials to contact with the SiH semiconductor, enabling electronic devices with high charge injection efficiency. Furthermore, the tunneling resistivity of all the MX_2_/SiH (M = Nb, Ta; X = S, Se) MSHs is low, confirming that they exhibit high electron injection efficiency. Our findings underscore fundamental insights for the design of high-performance multifunctional Schottky devices based on the metal–semiconductor MX_2_/SiH heterostructures with ultra-low contact barriers.

## Conflicts of interest

There are no conflicts to declare.

## Supplementary Material

NA-006-D4NA00482E-s001

## Data Availability

The data that support the findings of this study are available from the corresponding author upon reasonable request.
