# Supplementary material for: Achieving ultra-low contact barriers in MX2/SiH (M = Nb, Ta; X = S, Se) metal–semiconductor heterostructures: first-principles prediction
Source: Nanoscale Adv. 2024 Jul 26;6(19):4900–6. doi: 10.1039/d4na00482e (PMC11317907; doi:10.1039/d4na00482e)
Supplement: NA-006-D4NA00482E-s001 [file NA-006-D4NA00482E-s001.pdf]

Supplementary Information (SI) for Nanoscale Advances.
This journal is © The Royal Society of Chemistry 2024

Supporting Information (SI)

Achieving Ultra-Low Contact Barriers in MX_2/SiH (M = Nb, Ta; X = S, Se) Metal-Semiconductor Heterostructures: First-Principles Prediction

S. T. Nguyen[1], Chuong V. Nguyen[2,†], Huynh V. Phuc[3,†], Nguyen N. Hieu[4,5], Cuong Q. Nguyen[4,5,†]

[1]Faculty of Electrical Engineering, Hanoi University of Industry, Hanoi 100000, Vietnam.
Email: nguyensontung@haui.edu.vn

[2]Department of Materials Science and Engineering, Le Quy Don Technical University, Hanoi 100000, Vietnam.
Email: chuong.vnguyen@lqdtu.edu.vn

[3]Division of Theoretical Physics, Dong Thap University, Cao Lanh 870000, Vietnam.
E-mail: hvphuc@dthu.edu.vn

[4]Institute of Research and Development, Duy Tan University, Da Nang 550000, Vietnam.
E-mail: nguyenquangcuong3@duytan.edu.vn

[5]Faculty of Natural Sciences, Duy Tan University, Da Nang 550000, Vietnam

† To whom correspondence should be addressed.

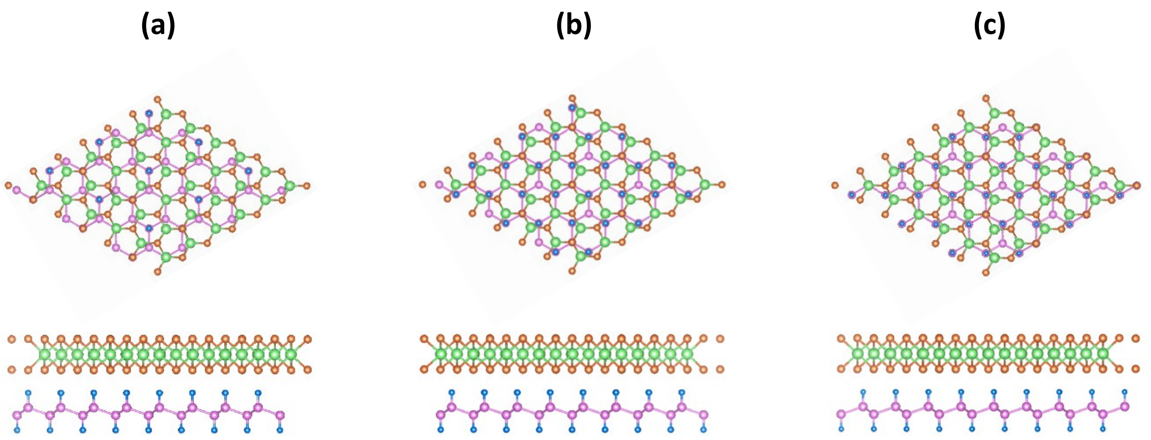

Figure S1. Atomic structures of the MX2/SiH heterostructures for three different stacking configurations of (a) SP-II, (b) SP-III and (c) SP-IV.

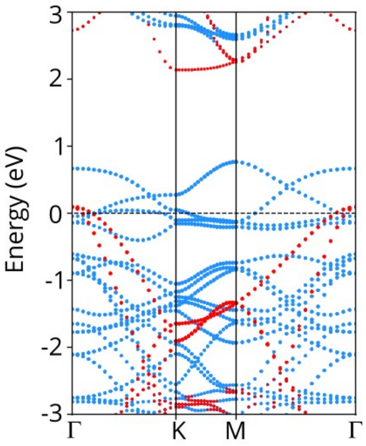

Fig. S2. Projected band structure of NbS2/SiH MHS under the compressive strain of ε_b = - 3 %.
